# Technological Barriers to Routine Genomic Surveillance for Vaccine Development Against SARS‐CoV‐2 in Africa: A Systematic Review

**DOI:** 10.1111/irv.70047

**Published:** 2024-11-18

**Authors:** Kimberly Cheryl Chido Konono, Keiko Msusa, Samuel Mpinganjira, Adidja Amani, Charles Nyagupe, Michael Ngigi

**Affiliations:** ^1^ Institute for Global Health University of Siena Siena SI Italy; ^2^ Global Clinical Development and Operations BioNTech SE Mainz Germany; ^3^ Clinical Operations TRIFT Alliance Ltd Kigali Rwanda; ^4^ National Microbiology Research Laboratory Harare Central Hospital Harare Zimbabwe

**Keywords:** Africa, COVID‐19, genomics, SARS‐CoV‐2, technological barriers, vaccine development

## Abstract

The Global Initiative on Sharing All Influenza Data, a public‐access database for sharing severe acute respiratory syndrome coronavirus 2 genomic sequencing data, has received significantly less data from African countries compared to the global total. Furthermore, the contribution of these data was infrequent and, for some countries, non‐existent. The primary aim of this review is to identify the technological barriers to routine genomic surveillance in Africa. PubMed and Google Scholar were searched for the relevant articles, and other eligible articles were identified from the reference list examination according to the PRISMA checklist. Eighty‐four full‐text articles were analysed for eligibility, and 49 published full‐texted articles were included in the final qualitative analysis. The main technological barriers identified were limited genomic surveillance capacity, limited genomic sequencing infrastructure, lack of resources and skilled or trained scientists, and the high cost of importing, establishing, and maintaining a genomic sequencing facility. The Africa Pathogen Genomics Initiative aims to improve genomic surveillance capacity across Africa, through resources, training, education, infrastructure, and regional sequencing centres. Furthermore, collaborations between African governments and international partners or national, private, and academic institutions are imperative to sustain genomic surveillance in Africa, and investment in genomic sequencing and research and development is paramount. Longer turnaround times interfere with global viral evolution monitoring and national implementation of effective policies to reduce the burden and disease. Establishing effective genomic surveillance systems guides public health responses and vaccine development for diseases endemic in Africa.

## 1 Introduction

The severe acute respiratory syndrome coronavirus 2 (SARS‐CoV‐2) pneumonia was detected in Wuhan, China, in December 2019, and the World Health Organization (WHO) declared it a pandemic in March 2020 [1]. According to the WHO, as of 11 August 2024, the virus has caused 776 million infections, and approximately 7.1 million deaths worldwide [2]. In Africa, 9.6 million infections and 175,526 deaths have been reported [2, 3].

SARS‐CoV‐2 is a betacoronavirus that belongs to the *Sarbecovirus* subgenus—one of the five subgenera under betacoronaviruses [4, 5]. It enters the host cell through the binding of the receptor‐binding domain (RBD) of the S protein to the angiotensin‐converting enzyme 2 (ACE‐2) receptor in the lungs and eventually to the other various organs; this process is mediated by the S1 subunit of the S protein [6, 7]. SARS‐CoV‐2 is a highly transmissible virus that has mutated, generating several more virulent variants that perpetuated the global pandemic, devastating economies as well as the health and education systems. As an RNA virus, SARS‐CoV‐2 is prone to replication errors that alter its genome through various nucleotide changes, allowing for the development of new variants [8].

Mutations on the S gene of the coronavirus often generate variants of concern (VOCs), such as the B.1.351 and B.1.1.7, variants in South Africa and the United Kingdom, respectively. VOCs may increase the transmission rate, re‐infection risk, severity of disease, viral replication, immune escape, and resistance to neutralising antibodies [9]. Unlike many other RNA viruses, SARS‐CoV‐2 is only able to attain approximately two polymorphisms per month, which slows the rate at which it mutates [8]. Mutations in various positions, such as the E484K mutation, may result in an increased ability to escape the host's immunity through the neutralisation of antibodies, leading to the reduced neutralising capacity of vaccines produced by Moderna and Pfizer [10].

From the original strain of the virus detected in Wuhan, China, the virus has undergone several evolutions producing the alpha (B.1.1.7), beta (B.1.351), gamma (P.1), delta (B.1.617) and omicron (B.1.1.529) VOCs, identified in the United Kingdom, South Africa, Brazil, India and South Africa, respectively [11, 12]. Additionally, as of January 2022, there was an increase in the sub‐lineages of Omicron, BA.1, BA.1.1, BA.2, BA.3, and BA.4, with BA.2 being more transmissible than BA.1 [12, 13]. As of 12 August 2024, there are no VOCs; however, there are five variants of interest (VOIs), namely, BA2.86, JN.1, XBB.1.5, EG.5, and XBB1.16, and a few variants under monitoring (VUMs) [14]. Most of the mutations on these viruses are on the S1 subunit, the target region for vaccines and monoclonal antibodies, and could contribute significantly to reduced vaccine effectiveness [11].

Viral evolution constantly occurs in unfavourable environments, and the emergence of new variants is a public health concern. Genomic sequencing is imperative in the early detection of VOCs or VOIs which may potentially, partially, or completely, evade the host's immune system [9]. The WHO defines genomic surveillance as the ‘constant monitoring and analysis of pathogens (bacteria, viruses, and parasites) and their genetic similarities and differences’. Genomic surveillance is required to track infectious diseases, develop new diagnostic tests, monitor changes in viral evolution, and identify new VOCs. It also allows for the implementation of public health interventions, social interventions, and vaccine and drug developments while controlling disease [15].

Genomic sequencing during the SARS‐CoV‐2 pandemic was utilised by scientists to characterise the virus, identify its variants and their prevalence in the population, explore the effect of therapeutics on the variants, and investigate viral transmission during outbreaks [16]. Furthermore, genomic sequencing is used to track the evolution of a pathogen—to identify drug resistance strains. Various technologies are utilised to characterise the virus, and these include the first‐generation sequencing technology such as the Sanger technology; the next‐generation sequencing (NGS) technology such as Illumina and Ion Torrent; second‐generation sequencing technology such as complete technology genomics; and the third‐generation sequencing technologies such as Oxford Nanopore Technology (ONT) and single molecule real‐time (SMRT).

Research groups have shared genomic sequences on public databases such as the Global Initiative on Sharing All Influenza Data (GISAID), allowing scientists from different countries to collaborate and track the virus' evolution [17]. By May 2020, Africa had only performed 186 SARS‐CoV‐2 genomic sequences out of the 30,000 performed globally, with eight countries contributing to the database [3]. According to GISAID, most African countries have contributed 0 (Eritrea and Mauritania) to 30,256 (South Africa) genomic sequences in the database [3]. Furthermore, 12 (South Africa) and 353 days (Somalia) were the longest time intervals reported, from the last date of submission among African countries [3]. As of 14 February 2022, out of 8,196,432 genomic sequences shared globally, Africa contributed 83,922 [3]. This reflects underlying challenges regarding the ability of most African countries to conduct genomic sequencing and to do so as frequently as required.

Thus, this review aimed to identify the technological barriers to routine genomic surveillance of SARS‐CoV‐2 in Africa.

## 2 Materials and Methods

A systematic literature review was conducted between July and August 2022 using the Preferred Reporting Items for Systematic Reviews and Meta‐Analysis (PRISMA) framework to identify research articles that address technological barriers to routine genomic surveillance of SARS‐CoV‐2 in Africa, the state of genomic surveillance and strategies used among African (or low‐and middle‐income) countries (LMICs) or cost‐effective solutions suggested to sustain and decentralise (where possible) genomic sequencing and surveillance of SARS‐CoV‐2.

The specific objectives were:

1. To highlight the current state of genomic surveillance for SARS‐CoV‐2 in LMICs and Africa as it relates to resources, infrastructure, technology, knowledge, collaborative partnerships, and cost and funding.
2. To determine the technological barriers (associated with the availability of resources, infrastructures, technology and platforms, knowledge, training opportunities, computational infrastructure, collaborative partnerships, and cost and funding), which may hinder routine and sustained genomic surveillance in Africa.
3. To determine the cost‐effective and technologically feasible ways of decentralising and sustaining genomic surveillance in Africa.

### 2.1 Search Strategy and Study Selection

Two major databases were searched, PubMed and Google Scholar, for freely available full‐text articles using the correct keywords from the objectives and the Boolean operators ‘AND’ or ‘OR’ as required: ((‘Genomic’) AND (‘Sequencing’ OR ‘Surveillance’), AND (‘SARS‐COV‐2’ OR ‘COVID’) AND (‘Africa’ OR ‘LMIC’ OR ‘challenges’ OR ‘barriers’ OR ‘limitations’ OR ‘decentralise’ OR ‘resources’ OR ‘solutions’ OR ‘strategies’ OR ‘implement’ OR ‘cost‐effective’ OR ‘technology’ OR ‘platforms’ OR ‘knowledge’ OR ‘infrastructure’ OR ‘collaboration’ OR ‘partnership’ OR ‘funding’ OR ‘training’ OR ‘cost’)) in the titles. Citations or records published from the year 2019 onwards and identified in each database were imported into a separate EndNote software version 20.4.1 library and sorted by alphabetical order.

Any duplicates identified were removed, as well as studies that were not relevant (after screening the titles and abstracts). Eligible articles were carefully screened against the inclusion and exclusion criteria and included in the final qualitative analysis. The reference lists of the articles obtained were also examined, and relevant articles were included in the final analysis. Reasons for the exclusion of any full‐text articles were documented and reported in Section 3.

### 2.2 Inclusion and Exclusion Criteria

This systematic review included full‐text articles that highlight, determine, or address the current state of genomic surveillance and the technological barriers of routine genomic surveillance related to the availability of adequate resources, infrastructures, technologies and platforms, knowledge, training opportunities, collaborative partnerships, and funding, as well as cost factors that may contribute to the unsustainability of genomic sequencing and surveillance in LMICs, particularly African countries. Additionally, studies highlighting cost‐effective solutions or strategies for sustaining routine genomic surveillance and decentralising genomic sequencing for SARS‐CoV‐2 in Africa were included. The review excluded studies that evaluate political and social barriers to sustaining routine genomic sequencing, studies of genomic sequencing/surveillance of other pathogens, and those that do not address the African or LMIC setting. The PICOs elements in Table 1 were utilised.

**TABLE 1:** The PICOs elements used to identify eligible studies to include in the systematic review.

Population	Genomic sequencing and/or surveillance studies for SARS‐COV‐2 in humans
Intervention	Genomic surveillance or sequencing for SARS‐COV‐2 studies conducted in/for Africa/LMICs/low‐resource settings
Comparison	None
Outcome	Current state and barriers of and solutions for genomic surveillance or sequencing in Africa

Abbreviations: LMICs, low‐ and middle‐income countries; SARS‐CoV‐2, severe acute respiratory syndrome coronavirus 2.

### 2.3 Data Extraction and Quality Assessment

The principal investigator independently performed the database search, study selection, and data extraction. The data extraction process used a Microsoft Excel template, and data are presented in tabular format. The study used the PRISMA checklist to transparently and effectively report the evidence obtained [18]. There was no risk of bias and quality assessment planned.

### 2.4 Statistical Analysis

Descriptive statistics (in counts, frequency, and percentage statistics where the unit of analysis will be the individual study) was used to summarise the data obtained from the studies in tabular or graphical format. Extracted data were utilised to summarise the barriers to routine genomic surveillance or sequencing, the current state of genomic surveillance in Africa, and effective strategies that could be implemented to sustain genomic sequencing in low‐resource settings. No subgroup analysis, sensitivity, and publication bias were performed. Effect models, effect measures, heterogeneity, and other quantitative analyses are not valid in this review.

## 3 Results

**FIGURE 1:**
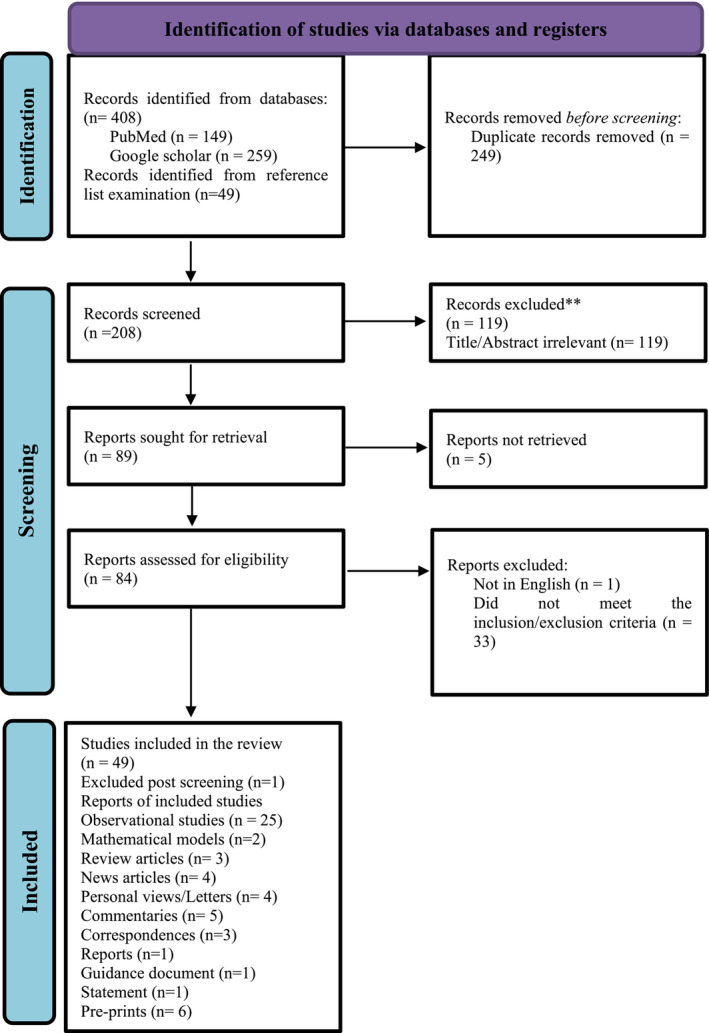
Flow diagram of the inclusion criteria of studies eligible for systematic review.

After the removal of duplicates, and the initial exclusion of all titles and abstracts that did not match the pre‐determined inclusion criteria, 84 full‐text articles were analysed for eligibility. Forty‐nine published full‐texted articles were included in the final qualitative analysis (Figure 1).

### 3.1 A Summary of the Reviewed Surveillance and Sequencing Articles

All studies included in the analysis are listed in Table 2. Studies primarily conducted in African countries accounted for 57.14% (*n* = 28) of the included articles. Additionally, the review included 12 studies conducted from a global perspective (24.50%) and 11 conducted in other LMICs (countries with a gross national income [GNI] per capita of ≤ $1085 to $4255; according to the World Bank classification) (22.45%), however containing data for resource‐limited settings (settings with inadequate health resources and healthcare systems according to global standards). Nineteen (38.78%) of the studies were conducted in South Africa (*n* = 8), Nigeria (*n* = 6) and Zimbabwe (*n* = 5).

**TABLE 2:** Characteristics of the included studies on genomic surveillance and sequencing of SARS‐CoV‐2.

No.	Author, year	Title of the study	Study setting	Data conducted for which setting	Method of study
1	Adepoju, 2021 [19]	Challenges of SARS‐CoV‐2 Genomic Surveillance in Africa	Nigeria	Africa	News article
2	Al Kalamouni, 2023 [20]	Genomic Surveillance of SARS‐CoV‐2 in COVID‐19 Vaccinated Healthcare Workers in Lebanon	Lebanon	Lebanon	Observational, genomic surveillance
3	Bahouq, 2021 [21]	Overview of Genomic Surveillance Related to Severe Acute Respiratory Syndrome Coronavirus 2 (SARS‐CoV‐2)	Morocco	Morocco	Review article
4	Brito, 2022 [22]	Global Disparities in SARS‐CoV‐2 Genomic Surveillance	United States	Global	Theoretical‐mathematical model
5	Butera, 2022 [23]	SARS‐CoV‐2 Genomic Surveillance in Rwanda: Introductions and Local Transmission of the B.1.617.2 (Delta) Variant of Concern	Rwanda	Rwanda	Observational, epidemiological survey—genomic analysis; a follow‐up study *[pre‐print]*
6	Butera, 2021 [24]	Genomic Sequencing of SARS‐CoV‐2 in Rwanda Reveals the Importance of Incoming Travelers on Lineage Diversity	Rwanda	Rwanda	Observational, epidemiological survey; genomic analysis
7	Chen, 2022 [25]	Global Landscape of SARS‐CoV‐2 Genomic Surveillance and Data Sharing	China	Global	Observational, global landscape analysis
8	Cyranoski, 2021 [26]	Alarming COVID Variants Show the Vital Role of Genomic Surveillance	Global	Global	News article
9	Dzinamarira, 2021 [27]	Insights From Zimbabwe's SARS‐CoV‐2 Genomic Surveillance	Zimbabwe	Zimbabwe	Commentary
10	Furuse, 2021 [28]	Genomic Sequencing Effort for SARS‐CoV‐2 by Country During the Pandemic	Japan	Global	Observational analysis
11	Han, 2022 [29]	Low Testing Rates Limit the Ability of Genomic Surveillance Programs to Monitor SARS‐CoV‐2 Variants: A Mathematical Modelling Study	Netherlands	LMICs	Theoretical‐mathematical model *[pre‐print]*
12	Harsha, 2022 [30]	The Role of SARS‐CoV‐2 Genomic Surveillance and Innovative Analytical Platforms for Informing Public Health Preparedness in Bengaluru, India	India	LMICs	Observational *[pre‐print]*
13	Hosch, 2021 [31]	Genomic Surveillance Enables the Identification of Co‐infections With Multiple SARS‐CoV‐2 Lineages in Equatorial Guinea	Equatorial Guinea	Central Africa	Observational surveillance
14	Kalia, 2021 [32]	The Lag in SARS‐CoV‐2 Genome Submissions to GISAID	India	Global	Correspondence
15	Kuja, 2022 [33]	Genomic Surveillance of SARS‐COV‐2 Reveals Diverse Circulating Variant Lineages in Nairobi and Kiambu County, Kenya	Kenya	Kenya	Observational, genomic epidemiology; phylogenome‐temporal analysis
16	Mahanta, 2022 [34]	Are Countries Becoming Better at SARS‐CoV‐2 Genomic Surveillance?	India	Global	Observational, global analysis
17	Malick, 2021 [35]	The Genomic Landscape of SARS‐CoV‐2: Surveillance of Variants of Concern	United States	Global	Review article
18	Mashe, 2021 [36]	Genomic Epidemiology and the Role of International and Regional Travel in the SARS‐CoV‐2 Epidemic in Zimbabwe: A Retrospective Study of Routinely Collected Surveillance Data	Zimbabwe	Zimbabwe	Observational, retrospective
19	Menon, 2021 [37]	Genomic Sequence of Worldwide Strains of SARS‐CoV‐2: Insights on the Role of Variants in Disease Epidemiology	India	Global	Observational
20	Merhi, 2022 [38]	SARS‐CoV‐2 Genomic Epidemiology: Data and Sequencing Infrastructure	Lebanon	LMICs	Observational, cost analysis
21	Murewanhema, 2021 [39]	Enhancing SARS‐CoV‐2 Surveillance Through Regular Genomic Sequencing Is an Essential Element of COVID‐19 Control in Resource‐Limited settings	Zimbabwe	Resource limited	Letter to the editor
22	Napit, 2023 [40]	Rapid Genomic Surveillance of SARS‐CoV‐2 in a Dense Urban Community of Kathmandu Valley Using Sewage Samples	Nepal	Resource limited	Observational, cross‐sectional
23	Ntoumi, 2021 [41]	Genomic Surveillance of SARS‐CoV‐2 in the Republic of Congo	DRC	Central Africa	Observational, epidemiological survey
24	Ortiz‐Pineda, 2022 [42]	Evolutionary Traits and Genomic Surveillance of SARS‐CoV‐2 in South America	Columbia	LICs	Review article
25	Pisano, 2022 [43]	SARS‐CoV‐2 Genomic Surveillance Enables the Identification of Delta/Omicron Co‐infections in Argentina	Argentina	Resource limited	Observational, case study
26	Robishaw, 2021 [8]	Genomic Surveillance to Combat COVID‐19: Challenges and Opportunities	United States	Global	Personal view
27	Romano, 2021 [44]	Genomic Surveillance of SARS‐CoV‐2: A Race Against Time	Brazil	Global	Commentary
28	Salles, 2022 [45]	Genomic Surveillance of SARS‐CoV‐2 Spike Gene by Sanger Sequencing	Brazil	Developing countries	Observational, laboratory protocol
29	Shaibu, 2021 [46]	Full‐Length Genomic Sanger Sequencing and Phylogenetic Analysis of Severe Acute Respiratory Syndrome Coronavirus 2 (SARS‐CoV‐2) in Nigeria	Nigeria	Resource limited	Observational, genomic sequencing and phylogenetic analysis
30	Souho, 2022 [47]	Study of the SARS‐CoV‐2 Genomic Data Generation to Evaluate the Introduction of Genomics in Epidemiological Surveillance and Public Health Decision‐Making	Togo	Africa	Observational, analytical study
31	Tegally, 2022 [48]	The Evolving SARS‐CoV‐2 Epidemic in Africa: Insights From Rapidly Expanding Genomic Surveillance	South Africa	Africa	Observational, epidemiological and phylogenetic analysis
32	Wilkinson, 2021 [49]	A Year of Genomic Surveillance Reveals How the SARS‐CoV‐2 Pandemic Unfolded in Africa	South Africa	Africa	Observational, phylogenetic and phylogeographic analyses
33	Chen, 2022 [50]	Global landscape of SARS‐COV‐2 genomic surveillance and data sharing	United States	Global	Observational, global analysis
34	Adepoju, 2022 [51]	African Coronavirus Surveillance Network Provides Early Warning for World	Nigeria	Africa	News article
35	Andersen, 2021 [52]	Variants in Africa: Recommendations for Preventing an Enduring Pandemic	England and Wales	Africa	Tony Blair Institute for Global Change report
36	Dzobo, 2021 [53]	Inadequate SARS‐CoV‐2 Genetic Sequencing Capacity in Zimbabwe: A Call to Urgently Address This Key Gap to Control Current and Future Waves	Zimbabwe	Zimbabwe	Perspective/letter
37	Giandhari, 2021 [54]	Early Transmission of SARS‐CoV‐2 in South Africa: An Epidemiological and Phylogenetic Report	South Africa	South Africa	Observational, molecular epidemiology study
38	Inzaule, 2021 [55]	Genomic‐Informed Pathogen Surveillance in Africa: Opportunities and Challenges	Ethiopia	Africa	Personal view
39	Makoni, 2020 [56]	Africa's $100‐million Pathogen Genomics Initiative	South Africa	Africa	News article
40	Mashe, 2021 [57]	Surveillance of SARS‐CoV‐2 in Zimbabwe Shows Dominance of Variants of Concern	Zimbabwe	LMICs	Correspondence
41	Mboowa, 2021 [58]	Whole‐Genome Sequencing of SARS‐CoV‐2 in Uganda: Implementation of the Low‐Cost ARTIC Protocol in Resource‐Limited Settings	Uganda	LMICs	Observational, cross‐sectional
42	Msomi, 2020 [59]	A Genomics Network Established to Respond Rapidly to Public Health Threats in South Africa	South Africa	South Africa	Commentary
43	NCDC, 2021 [60]	Statement on Variants of SARS‐COV‐2 in Nigeria	Nigeria	Nigeria	Nigeria Centre for Disease Control statement
44	Onwuamah, 2021 [61]	SARS‐CoV‐2 Sequencing Collaboration in West Africa Shows Best Practices	Nigeria	West Africa	Commentary
45	Otu, 2021 [62]	Africa Needs More Genome Sequencing to Tackle New Variants of SARS‐CoV‐2	Nigeria	Africa	Correspondence
46	Pillay, 2020 [63]	Whole Genome Sequencing of SARS‐CoV‐2: Adapting Illumina Protocols for Quick and Accurate Outbreak Investigation During a Pandemic	South Africa	South Africa	Observational, phylogenetic analysis
47	Tessema, 2020 [64]	Accelerating Genomics‐Based Surveillance for COVID‐19 Response in Africa	Africa	Africa	Commentary
48	Viana, 2022 [65]	Rapid Epidemic Expansion of the SARS‐CoV‐2 Omicron Variant in Southern Africa	Botswana and South Africa	Southern Africa	Observational, molecular epidemiology
49	WHO, 2021 [66]	Guidance for Surveillance of SARS‐CoV‐2 Variants: Interim Guidance, 9 August 2021	Switzerland	Global	WHO interim guidance

Abbreviations: COVID‐19, coronavirus disease 2019; SARS‐COV‐2, severe acute respiratory syndrome coronavirus 2 (SARS‐COV‐2); WHO, World Health Organization.

### 3.2 Ability to Conduct Genomic Sequences

There are 24 African countries reported with the capacity to conduct genomic sequences for COVID‐19 and submit genomes to GISAID, with varying strengths of genomic surveillance systems [48]. The sequencing capacities of South Africa, Nigeria, Kenya, the Gambia, the DRC, and Zimbabwe were the most frequently mentioned in the included articles.

### 3.3 Genomic Surveillance/Sequencing Systems in Africa

#### 3.3.1 Network for Genomic Surveillance in South Africa (NGS‐SA)

The Network for Genomic Surveillance in South Africa (NGS‐SA) established in 2020 [58] comprises public universities and government laboratories that sequence SARS‐CoV‐2. NGS‐SA deposits data into a sequencing read archive weekly and submits these genomes to GISAID. Collaborations with the South African National Bioinformatics Institute ensure that genome data produced and analysed in South Africa are of adequate quality [19, 26, 49, 54, 59, 65].

Overall, the NGS‐SA has seven sequencing hubs that randomly receive positive COVID‐19 samples from private and government laboratories in South Africa [65]. Similar to the sequencing hub in Nigeria, the inactivation of the virus‐containing samples in a BSL III laboratory precedes the transportation of samples to a BSL II laboratory for sequencing [37, 46, 54].

#### 3.3.2 Nigeria's Genomic Surveillance Network

There are three institutions in Nigeria with the capacity to conduct genomic sequences, namely, the African Centre of Excellence for Genomics of Infectious Diseases (ACEGID), Nigeria Centres for Disease Control (NCDC), and the Nigeria Institute for Medical Research (NIMR). The first SARS‐CoV‐2 virus sequencing in Africa was conducted by the ACEGID within 72 h of identifying the virus within their borders [33, 49, 52, 60]. Additionally, there is a collaboration between the universities of Ibadan in Nigeria and north‐western in the United States that allows for SARS‐CoV‐2 viral sequencing [60].

#### 3.3.3 Zimbabwe's Genomic Sequencing Capacity

The National Microbiology Reference Laboratory (NMRL) is the only laboratory performing SARS‐CoV‐2 sequences for the entire country using only two ONT sequencers received from the Quadram Institute; however, there are many private and public laboratories with the capacity to diagnose COVID‐19 using polymerase chain reaction (PCR) tests [53]. The country's limited capacity and the goal of meeting the testing need calls for genomic sequencing supplementation by the Quadram Institute and the Kwazulu‐Natal Research Innovation and Sequencing Platform [53]. Furthermore, to boost sequencing capacity in the country, the Africa Centres for Disease Control and Prevention (Africa‐CDC) and Quadram Biosciences Institute (QIB, UK) provided Zimbabwe with training and funding, respectively [53]^.^

#### 3.3.4 Uganda's Genomic Surveillance Network

COVID‐19 diagnostic testing conducted primarily at the Makerere University Molecular Diagnostics Laboratory allows for archiving of samples at the Integrated Biorepository of H3Africa [58]. Sequencing of the virus and submission of the genomes to GISAID is conducted by the Uganda Virus Research Institute (UVRI) [58].

#### 3.3.5 Equatorial Guinea's Genomic Sequencing Capacity

The Public Health Laboratory on Bioko Island is the site for genomic surveillance, established as part of the country's response to the SARS‐CoV‐2 pandemic [31].

#### 3.3.6 The Gambia's Genomic Surveillance Network

The Genomics Unit of the Medical Research Council in Gambia (The Gambia‐MRCG) collaborates with the Centre for Human Virology and Genomics to sequence SARS‐CoV‐2 in the country as well as partners across West Africa [61].

#### 3.3.7 Botswana's Genomic Sequencing Capacity

Laboratories in both sectors (private and public) transport random samples to the National Health Laboratory (NHL) and the Botswana Harvard HIV Reference Laboratory (BHHRL) for genomic sequencing of positive COVID‐19 samples [65].

#### 3.3.8 Morocco's COVID‐19 Genomic Consortium

There is a COVID‐19 genomics consortium composed of a network of laboratories with sequencing platforms such as the Reference Laboratory for Influenza and Respiratory Viruses of the National Institute of Hygiene (INH), the Medical Biotechnology Laboratory (MedBiotech) of Faculty of Medicine and Pharmacy, the Functional Genomic Platform of the National Centre for Research Science and Technology (FGP‐CNRST) and the Institut Pasteur of the Morocco (IP Maroc) [21].

### 3.4 General Current State of the Genomic Surveillance and Sequencing Infrastructure in Africa/LMICs

Ten articles (20.4%) reported on the limited genomic surveillance capacity in Africa [25, 26, 29, 35, 40, 44, 48, 50, 53, 64]. But five articles (10.2%) emphasised the disproportionate distribution of genomic sequencing capacity across the globe and within Africa [19, 31, 45, 52, 65]. Additionally, there is a higher sequencing ratio per reported case observed in high‐income countries (HICs) compared to LMICs [22, 25, 29, 36, 50, 52]. On the other hand, four articles (8.2%) applauded the obvious improvements in genomic surveillance on the continent, particularly in building capacity and integrating genomic data into public health responses [34, 47, 48, 51].

Sixteen of the 54 African countries remain with no genomic sequencing capacity within their borders, and in countries with capacity, the majority of it is concentrated in private laboratories [48, 52, 55, 56]. Regarding submission of genomic sequences to public databases, although three articles reported longer turnaround times from LMICs including African countries [32, 49, 51], another three articles acknowledged the improved turnaround times from African countries [25, 48, 51], such that the shortest turnaround time in the best facilities in Africa is now 6 days compared to the previous time of 19 days [25, 32, 49, 51, 64]. In Africa, the Gambia, Nigeria, Mauritius, Reunion, DRC, Senegal, Kenya, Mayotte, South Africa, and Djibouti have a higher proportion of sequences per reported case as indicated in seven articles [22, 25, 28, 47, 49, 50, 52].

Twelve of the articles reported the use of Illumina technology in 10 LMICs, followed by the use of ONT in eight LMICs. Overall, a significant percentage of the next‐generation sequencers on the continent are in South Africa, Kenya, Egypt, Nigeria, and Morocco [52, 55]. Approximately one‐third (16/49) of the articles mentioned whole genome sequencing (WGS) as the type of genomic sequencing performed in Africa or other LMICs, specifically countries such as Kenya, Rwanda, Equatorial Guinea, Zimbabwe, Nepal, Lebanon, Argentina, Nigeria, South Africa, DRC, Uganda and the Gambia [20, 23, 24, 31, 33, 36, 37, 40, 41, 43, 46, 54, 58, 61, 63, 65]. A significant number of these countries use the ARTIC protocol for viral amplification as a PCR amplicon‐based method [20, 23, 24, 31, 33, 36, 38, 40, 41, 54, 58, 61, 63].

### 3.5 Technological Barriers of Genomic Surveillance and Sequencing in Africa/LMICs/Low‐Resource Settings

The primary reason for the lack of genomic surveillance data in LMICs including several African countries is limitations in infrastructure capacity [19, 25, 35, 42, 48, 49, 53, 58]. The second most reported barrier to sustaining genomic surveillance infrastructure in Africa is the lack of access to reference sequencing laboratories and the lack of any or adequately established facilities available within the country or continent to meet all sequencing requirements [25, 38, 40, 47, 54, 58]. Other barriers include low Internet speed, inadequate investment in research and development, lack of prioritisation of genomic surveillance attributable to underdeveloped public health systems, sample degradation, and challenges with storage of samples [32, 38, 39, 61, 63].

The most reported technological barrier regarding resources is the insufficient number of trained scientists or skilled personnel available in African countries to perform genomic sequences, analyse genomic data (bioinformatics), and bridge the gap between genomic data and public health interventions or policies [19, 29, 30, 38, 42, 47, 49, 53, 55, 56, 58]. Additionally, lack of resources [25, 26, 40, 46, 63, 64], lack of reagents owing to disruptions in global supply chains [49, 54, 58, 63] and shipment delays exacerbated by border closers and travel restrictions [22, 52, 61, 63] were major disruptions to sustained genomic surveillance during the pandemic.

Furthermore, three articles reported limitations in access to sequencing machinery, technology, and tools required to perform genomic‐based detection and surveillance [29, 45, 47]. Another four articles highlighted the additional costs incurred by LMICs when importing equipment and other consumables, which account for shipping, customs, and local supplier profit margin costs [22, 38, 55, 63]. Six articles emphasised the high cost associated with running an NGS facility, even in a developed country, and of these articles, one stated the start‐up cost of establishing such a facility, to be above $100,000, not exceeding $700,000 [38, 40, 45, 47, 52, 55]. The lack of funding was highlighted in four articles [32, 38, 47, 52], and this was specific to laboratory and surveillance infrastructures and in national health, research, and development, which affects the ability of countries to scale up genomic surveillance [22, 29].

### 3.6 Strategies Implemented by Other African Countries to Sustain and Decentralise Genomic Surveillance

One of the most important strategies implemented on the continent is the investment in capacity building in Africa through the collaboration of Africa‐CDC and the WHO AFRO, as well as other African nations and international partners [48, 51, 52, 53, 64]. The Africa Pathogen Genomics Initiative (Africa‐PGI) aims to strengthen the genomic sequencing capacity of 20 countries in Africa, utilising funding received from various partners in October 2020 [51, 56]. The African genome sequencing laboratory network's objective is to ensure that laboratories with sequencing capacity also receive and process samples from countries that are completely (i.e., Namibia, Angola, and South Sudan) or partially incapacitated [19, 48, 50, 53, 55, 58]. The regional reference sequencing centres are located in Nigeria, Kenya, South Africa, Senegal, Ghana, DRC, and Uganda [51, 54, 60, 62]. Alternatively, other countries, particularly in the western and northern parts of Africa, use facilities outside of Africa to supplement their sequencing efforts [48].

### 3.7 Cost‐Effective Solutions for Sustaining Genomic Surveillance in Africa

The majority of the articles recommend strengthening or initiating collaborations and partnerships between African countries and research institutions in HICs, national public health institutions, and academic institutions as well as laboratories, both private and government [8, 22, 28, 39, 48, 52, 53, 56, 58, 59, 62, 67]. Five articles emphasised the advantages of ONT over other technologies, owing to its affordability, portability, reliability, proven benefit in real‐time outbreak surveillance, and shorter turnaround time [38, 40, 53, 56, 61]. Additionally, four articles suggested targeted sampling strategies as opposed to representative strategies for low‐resource settings [31, 35, 48, 59].

### 3.8 Cost‐Effective Solutions for Decentralising Genomic Surveillance in Africa

The most reported cost‐effective solution is the use of continental efforts such as the Africa‐CDC and Pan American Health Organization (PAHO) networks to build and sustain genomic surveillance and research in low‐resource settings, particularly in countries with no capacity for it [22, 29, 44]. Other solutions include establishing quality testing and analysis standards across the continent, disease‐specific standardised tools, and the use of environmental surveillance instead of clinical surveillance in low‐resource settings [40, 55].

Table 3 summarises the main technological barriers and implemented strategies or cost‐effective solutions suggested in Africa.

**TABLE 3:** The main technological barriers and implemented strategies or cost‐effective solutions identified in this review.

Technological barriers	Cost‐effective solutions or strategies implemented
Limited genomic sequencing infrastructure	Capacity building across Africa through the Africa‐CDC and Africa‐PGI Consider multi‐pathogen core sequencing laboratories vs. multiple single‐pathogen sequencing laboratories
Limited access to sequencing facilities or available established facilities	They have established regional sequencing centres in seven African countries as part of the African genomic sequencing laboratory network Use of sequencing facilities outside of Africa
Lack of or inadequate diagnostic capacity for COVID‐19 disease	
Lack of skilled personnel‐ Challenges in training and retaining the required personnel	Training of scientists by the Africa‐PGI and other institutions in and outside of Africa, as supported by African governments Regional workshops in bioinformatics and genomics Integrate these fields into existing university or college course
Lack of resources and sequencing tools and equipment	Sequencing a few samples but consistently Targeted sampling strategies versus representative sampling
Use of the ARTIC protocol is laborious and lengthy	Modifying existing protocols to reduce processing time
Conducting WGS is expensive, time‐consuming, requires skilled personnel and specific equipment	Hybrid approach of WGS and RT‐qPCR assays
Limited resources dedicated to R&D and genomics	Investments into R&D and genomic surveillance by African governments
Not enough scientific knowledge to guide public health (PH) responses	Development of systems to pre‐empt pandemics, standardise bioinformatic tools and integrate pathogen genomics into PH responses
Lack of cooperative support from international, regional, and public health groups	Strengthen national collaborations between private, public and academic institutions and other international institutions or advanced sequencing centres
Additional costs, such as shipping, increase the cost of imported resources	Regional or continental bulk purchasing
The cost of establishing and running a sequencing facility is high	Use of portable and field deployable platforms, that is, ONT Multiplexing reduces the cost per sample
The cost of reagents is higher in LMICs than HICs	Price negotiations with the manufacturer
Lack of funding	The various partners invested 100 million into the Africa‐PGI Include genomic sequencing as part of the emergency response system for COVID‐19 Use the WHO shipping fund to transport samples to regional sequencing centres
It is challenging to attain the needed computational infrastructure to process genomic data	Computers with graphical processing units Hybrid approach of cloud‐based and on‐site computing infrastructure

Abbreviations: Africa‐CDC, Africa Centres for Disease Control and Prevention; Africa‐PGI, African Pathogen Genomics Initiative; COVID‐19, coronavirus disease 2019; HIC, high‐income countries; LMICs, low‐ and middle‐income countries; ONT, Oxford Nanopore Technology; R&D, research and development; RT‐PCR, reverse transcriptase polymerase chain reaction; WGS, whole genome sequencing; WHO, World Health Organization.

## 4 Discussion

To our knowledge, this is the first systematic review of the barriers and current state of genomic surveillance of SARS‐CoV‐2 in Africa. The findings of this research focused on three areas: the current state of genomic sequencing and surveillance, technological barriers and strategies implemented by other African or low‐resource settings, and cost‐effective solutions applicable to this setting.

The genomic surveillance systems in Africa continue to improve, facilitated primarily by the Africa‐PGI, as one example of how large‐scale surveillance system deficiencies have been addressed [34, 47, 48, 51].

In 2019 when the pandemic began, the majority of the countries had no genomic surveillance capacity, which is in contrast to the United Kingdom, which had the capacity prior, although genomic sequencing was not a standard public health response until the COVID‐19 pandemic [68]. Countries in Africa established these systems in 2020 during the pandemic as they discovered the importance of pathogen genomics and gained support to do so from international, academic, and public health institutions in and outside of Africa [32]. Contrarily, the United Kingdom and the United States built on their previously established genomic sequencing infrastructure to adopt a centralised genomic surveillance system for SARS‐CoV‐2 on a larger scale to cope with the number of samples for sequencing to meet the weekly targets [68, 69].

Scientists in African countries required training to use and analyse the next‐generation sequencers and genomic data generated, and in contrast, the United Kingdom already had skilled personnel working in these scientific fields [68]. Resources, reagents, and equipment were challenging to obtain in Africa due to travel restrictions and reduced global supply chains, and the COVID‐19 Genomics UK (COG‐UK) consortium faced similar challenges attributed to disruptions in the global supply chain [49, 58, 70]. Furthermore, a significant number of African countries require external funding to sustain genomic surveillance as health budgets are limited for this purpose and are mostly directed towards testing, treating, vaccines and vaccination, contact tracing, and other activities that are imperative during a pandemic [19, 61].

The PCR amplicon‐based method utilised by the majority of the African countries has been beneficial during the SARS‐CoV‐2 pandemic, as the virus has a low genetic diversity and the method is cheaper, faster, highly sensitive, and specific [71, 72, 73]. Additionally, it is preferred in genomic screening for virulence, drug resistance viral strains and clinical diagnosis of not only SARS‐CoV‐2 but HIV as well as other bacteria, fungi, and respiratory viruses [72]. The increased genomic sequencing capacity in Africa requires a larger computational infrastructure for data processing, storage, and analysis; the lack of this infrastructure has become more evident and is worsened by Internet access challenges in various regions of Africa [21, 38, 61]. Hence, the need to build human capacity must be complemented with efforts to acquire sustainable equipment infrastructure.

The Africa‐PGI was established to address the numerous gaps in the genomic surveillance systems in Africa related to infrastructure, resources, training and knowledge, computational infrastructure, and use of genomic data to inform public health policy in various African countries [19, 52, 53, 56, 64, 74]. This African genomic sequencing laboratory network aims to identify and inform public health responses to COVID‐19 and other diseases such as HIV/AIDS, malaria, tuberculosis, and cholera which are endemic in Africa, as well as other epidemic threats, such as influenza A/B and respiratory syncytial virus (RSV) A/B is imperative [56]. Additionally, training scientists in NGS technology use is one of the core goals, and countries such as Zimbabwe have already benefited from it [53]^.^

Among many others, the COG‐UK consortium, established in March 2020, has a decentralised network with regional sequencing centres in academic and public institutions, similar to that of the Africa‐PGI and its network of sequencing laboratories [75]. As an example of an established surveillance system in an HIC, whose model could be compared to that of the Africa‐PGI, it is involved in genomic sequencing and data analysis, research, training, and increasing global access to genomic data [68]. Both these networks required extensive funding to be established.

Likewise, the genomic sequences submitted to GISAID by South Africa have been the most in number amongst all African countries (approximately 50% of all submitted sequences), with private laboratories, public laboratories, and academic institutions working together in sequencing, diagnosis (these laboratories submit samples randomly to sequencing laboratories) and bioinformatic analysis of SARS‐CoV‐2 in South Africa [49, 51, 55, 59, 65]. Countries such as Zimbabwe have one public laboratory conducting genomic sequences for the whole country. It is insufficient and not adequate to meet the required sample size per week, hence the use of sequencing centres in other countries to meet this need [53]. Contrarily, within the COG‐UK consortium, the genomic data are primarily generated by a not‐for‐profit research institute and public health institutions [68].

Pillay et al. and Pisano et al. reported the arduous and extended process associated with the use of the ARTIC protocol for genomic sequencing, in addition to the extensive resources, training, and time required to conduct WGS [43, 63]. In countries such as Uganda, Equatorial Guinea, and Kenya, RT‐qPCR assays have been used alone or prior to WGS to reduce the cost and time while increasing the scope of coverage [31, 33, 58, 66]. Furthermore, to conduct genomic sequencing of SARS‐CoV‐2 in a manner that provides relevant data in real‐time, consistent sequencing of small samples is more beneficial for low‐resource settings, by adopting a target‐based and not a population‐based sequencing strategy, as observed in South Africa and Nigeria [26, 31, 48, 59].

Though the disadvantages of the ARTIC protocol were noted, it is cheaper, more sensitive, and easier to scale up [58]. Similarly, HICs have limited resources, and thus, the sampling strategies employed must maximise the efficient use of the resources available. The COG‐UK consortium's approach to sampling is to include population and target‐based sampling strategies while prioritising local samples as necessary [75]. Furthermore, a wide range of sequencing methods or approaches including amplicon and NGS technologies are all used interchangeably [75].

Comparing the Sanger technology, a first‐generation sequencer, to NGS technology, there is a preference for ONT in LMICs, particularly the MinION platform [38, 40, 53, 56, 61]. The benefits of this platform were noted during the previous outbreaks in Africa due to Lassa fever and Ebola. However, for countries without access to NGS technologies, the Sanger technology is still easily accessible and highly accurate [45, 46].

## 5 Limitations

Information regarding the specific genomic surveillance systems and an accurate number of sequences uploaded to GISAID from most African countries is unavailable primarily due to a lack of published data/articles; this has introduced information bias within the study. Furthermore, failure to retrieve five articles that were not freely available is associated with information bias. The quality of the articles included in this review was not assessed, and low methodological quality was not a criterion for exclusion, resulting in the inclusion of a significant number of articles of low grade of evidence. However, all articles included provided valuable insight into the study objectives. No authors were contacted for more information to enable an accurate assessment of the quality of each study's methodology; however, this does not affect the study findings as the study quality or the grade of evidence was not assessed for the articles included in the review. Information regarding SARS‐CoV‐2 rapidly changes and using information in studies published in 2020 and 2021 is not an accurate representation of the study objectives. However, one can compile the data in chronological order to understand the trends. Lastly, due to the extensive nature of the review, and the high volume of information sourced, the possibility of selection and information bias is high, as only the primary investigator selected the studies and extracted the data in this study.

## 6 Conclusions

Genomic surveillance is imperative for pathogen evolution and monitoring, not only for SARS‐CoV‐2 but also for various other pathogens that plague the African continent, causing frequent outbreaks. The findings of this review reinforce the importance of decentralising genomic sequencing of pathogens in Africa. Every country should be able to conduct genomic sequences and provide basic bioinformatic analysis of genomic data to the public health authorities for policy decision‐making. Furthermore, with the improvement of genomic surveillance systems in Africa, policies are required to protect individual patient data and standardise bioinformatic and genomic sequencing protocols.

A fully functional genomic surveillance system requires adequate and continuous investment in genomic sequencing infrastructure, resources and teaching, research, and development. African governments need to focus on facilitating and investing in research, not only in pathogen genomics but also in vaccine development for COVID‐19, HIV, tuberculosis, influenza and RSV‐associated respiratory illnesses, and other endemic diseases.

Due to the limitations in genomic sequencing infrastructure, submission of SARS‐CoV‐2 genomic data to public databases is inconsistent and associated with long turnaround times. From a global health perspective, this interferes with the monitoring of the virus' evolution, leading to the emergence of VOCs and delayed implementation of public health interventions, including effective vaccination programmes. Furthermore, nationally, real‐time genomic sequencing ensures that policies implemented are effective in reducing the burden of infection and disease, and the burden on the healthcare systems, particularly in African countries where the poor healthcare systems are struggling to manage endemic diseases. Establishing a robust genomic surveillance system and a national genomic sequencing network by collaborating with private laboratories and academic institutions is a long‐term strategy, as detecting seasonal changes in circulating respiratory virus strains informs public health responses and guides vaccine development for not only SARS‐CoV‐2 but other respiratory viruses such as influenza and RSV, in addition to diseases endemic in the African region.

## Author Contributions

**Kimberly Cheryl Chido Konono:** conceptualisation (lead), writing – original draft (lead), formal analysis (lead), writing – review and editing (equal); methodology (equal), investigation (lead), visualisation (lead). **Keiko Msusa:** conceptualisation (support), methodology (equal), supervision (lead), project administration (lead), writing – review and editing (equal), visualisation (supporting). **Samuel Mpinganjira:** review and editing (equal). **Adidja Amani:** writing – review and editing (equal). **Charles Nyagupe:** review and editing (equal). **Michael Ngigi:** review and editing (equal).

## Ethics Statement

The protocol for the research project was approved by the University of Siena's Research Ethics Committee, and it conforms to the provisions of the Declaration of Helsinki in 1995 (as revised in Fortaleza, Brazil, October 2013).

## Consent

Informed consent was waived for this systematic review.

## Conflicts of Interest

The authors declare no conflicts of interest.

### Peer Review

The peer review history for this article is available at https://www.webofscience.com/api/gateway/wos/peer‐review/10.1111/irv.70047.

## Data Availability

Data are available from the corresponding author upon request.
